# Knowledge, attitude towards, and utilization of friendly health services among school adolescents in the pastoral community of Guji zone, Ethiopia: an institution-based comparative cross-sectional study

**DOI:** 10.3389/frph.2024.1291742

**Published:** 2024-10-02

**Authors:** Gobena Godana, Silesh Garoma, Nicola Ayers, Muluembet Abera

**Affiliations:** ^1^ School of Public Health, Department of Population and Family Health, Jimma, Ethiopia; ^2^ Department of Public Health of Adama Hospital Medical College, BPP University School of Nursing, London, United Kingdom; ^3^ Department of Nursing, Adama Hospital Medical College, Adama, Ethiopia

**Keywords:** sexual and reproductive health, adolescent, knowledge, attitude, utilization, pastoral-community

## Abstract

**Aim:**

Although sexual and reproductive health for adolescents is a recognized fundamental human right and a critical component of health policy, it is poorly addressed and seldom researched in pastoral communities. The study aimed to determine the status of sexual and reproductive health knowledge, attitudes, and practice among pastoral school adolescents in Ethiopia.

**Study design:**

An Institution-based comparative study was conducted from Nov. 2020 to Jan. 2021.

**Methods:**

We conducted a comparative cross-sectional study at four randomly selected high schools. Seven hundred seventy-three adolescent students participated, with 384 from Gorodola and 389 from Wadara districts, Guji zone Ethiopia. The data was collected using 34 self-administered questions and analyzed using descriptive, *t*-test, and linear regression models.

**Results:**

The study found that only 44.2% of all the participants had good knowledge, 46.1% had good attitudes and 35.4% had good utilization of Sexual and Reproductive Health Services. Respondents from Wadara High School had significantly higher mean knowledge scores (49.3% vs. 44.2%, *p* < 0.01) than those of Gorodola High School. There was no significant difference in mean utilization scores between case and compare (45.08% vs. 37%, *p* > 0.01). Adolescents who were not communicated on SRH matters, previously utilized FHS, and visited Friendly Health facilities were associated with poor utilization of sexual and reproductive health services.

**Conclusion and public health contributions:**

Wadera High School adolescents have better Sexual and reproductive health knowledge and utilization than Gorodola high schools. Community public health care providers in Wadara District explain the outcome through their contributions. Within the context of inherent disadvantage in the school environment setting, there is a need to improve sexual and reproductive health education with a greater emphasis on school girls.

## Introduction

Adolescence is a critical period in human development marked by rapid physical, psychosocial, intellectual, and emotional maturation as well as sexual and reproductive maturation. It is a nearly universal stage of the socialization cycle and a time for learning, exploring, and establishing healthy attitudes and behaviors for the future (1). The adolescent age group comprises 20% of the world population and 26.1% of the Ethiopian population (2). Despite the enormous adolescent population worldwide, providing appropriate, comprehensive Knowledge and utilization of sexual and reproductive health (SRH) at high school is critical for lifelong protective benefit (3). However, in Sub-Saharan African countries, including Ethiopia, SRH education is far from being institutionalized as in most developing countries, where the HIV epidemic, unsafe sex, and unsafe abortion are common.

Early adolescents in sub-Saharan countries are sexually active, although their reproductive health knowledge is deficient. Thus, only about 24.5% of in-school adolescents have comprehensive HIV/AIDS knowledge (4). In contradiction to this finding, in north Ethiopia, 88% of Mekelle high school students were highly knowledgeable. In Ethiopia, the perception of being at risk of acquiring HIV is only about 21.6%–24.5% of in-school adolescents (4–6). SRH education has improved SRH knowledge among adolescents in Riyadh-Saudi Arabia, Nigeria, and Vanuatu (7–9).

Adolescent-friendly health services (AFHS) are an evidence-based approach to reducing barriers to SRH service uptake. Similarly, a study conducted in Burkina Faso, Ethiopia, and Nigeria showed that unmarried adolescents utilized nearly 50% of Reproductive Health Services (10).

On the other hand, an assessment in Amhara demonstrated that only 41%–45% of adolescents had utilized sexual and reproductive health services (11, 12). A similar study conducted in Harar showed that about 64% of adolescents utilized friendly Health services at least once (13).

Public Health facilities were not selected as the first choice by adolescents due to providers’ judgment, privacy, and confidentiality dilemmas. Adolescent-friendly health service utilization in the Bale zone is 46.9% (14). However, there needs to be more evidence in Ethiopia on the knowledge, attitudes, and practices (KAP) level on SRH among pastoralist school adolescent populations. Pastoral communities often have unique cultural practices and beliefs. A KAP study adolescent Sexual and reproductive health helps in understanding these cultural nuances, ensuring that any interventions respect and align with local traditions and practices. As a result, the purpose of this study was to measure the Knowledge, Attitude and Practice (KAP) on the SRH and associated influencing factors among pastoral school adolescents in Guji zone, Ethiopia, and to use it as the foundation for, and the first to contribute to, improving the KAP of adolescents in the pastoral study area, Ethiopia.

## Methods and materials

### Study areas participants and design

An institution-based comparative cross-sectional study was conducted from November 2020 to January 2021. Our study population consisted of school adolescents aged 14–19 who attended a governmental high school in Ethiopia's Guji zone pastoral communities.

### Sample size

A probability sampling method was applied to select high schools in urban (Gorodola) and rural (Wadara) areas. For urban participants, two high schools (Gorodola High School) were chosen randomly from a list of high schools for the study. Similarly, we selected two Wadera High Schools for rural respondents. School Adolescents from the selected high schools took part in our study.

We calculated the required sample size for the first objective of this study using a single population proportion and the following assumptions. We calculated the required sample size for this study (focusing on factors associated with KAP) using the Stata12 software program, considering the following assumptions: We set the level of confidence (α) at 95%, the power at 90%, and the ratio at 46.5% unexposed to 64% exposed. exposed) is only used once [Mengistie et al. (15)], (13). Hence, the calculated sample size was 179 for each case and compared group. Hence, with the assumption of 2 as —the design effect and 10% as the non-respondent rate, the total sample size for the third objective is 788 (16). Finally, 788 respondents were included in our study and divided into four high schools, 394 from Gorodola and 394 from Wadera districts.

A multistage random sampling method was applied to select study participants. First, we randomly selected two high schools from a pool of 8 governmental high schools. Second, we equally assigned the total sample size (788) to each selected high school. Then, distribute to each section at random. Finally, a list of identification numbers was taken from the roster of each eligible adolescent student in the randomly selected schools (aged 14–19 years as a sampling frame) and distributed to each section to each school's administrators. We chose study participants using a simple random sampling technique using computer-generated random numbers. Before the data was collected, all schools agreed to participate after being informed of the study's purpose. In addition, before participating in the study, the selected adolescents and families were informed of the research intention and provided informed consent.

## Study variables and measurements

Participants’ knowledge of sexual and reproductive health (SRH) was assessed based on their ability to score above the mean in SRH information, attitudes, and service utilization. We measured SRH knowledge across three domains, including four items, such as whether participants had ever communicated about AFSRH information. Yes,/one had appropriate information or no/zero if not. We measured knowledge about friendly health services using two items, asking participants whether facilities provide friendly health services. Moreover, having appropriate Knowledge about SRH is measured with (2 items; e.g., unwanted pregnancy cannot happen with Single Sexual intercourse.) yes/one if had appropriate knowledge or no/zero if not.

### Attitudes

It refers to adolescents, parents, and community Health care providers’ thinking/feelings on friendly sexual and reproductive health services and practices, about their personal beliefs, values, and religions. We measured attitudes that promote SRH knowledge within two domains: Parents support their teens on SRH services with (2 items; e.g., Are you freely discussing SRH issues within your family? Compassionate care and attractive FHS (6 items, e.g., do healthcare providers are compassionate?) yes/one if enabling attitudes and no/zero if not.

### Perceptions

Perceptions were measured using five items (e.g., I seek friendly health information and services only when I face SRH problems). Agree/one if with appropriate perceptions and disagree/zero if not.

### Practice

**Figure 1:**
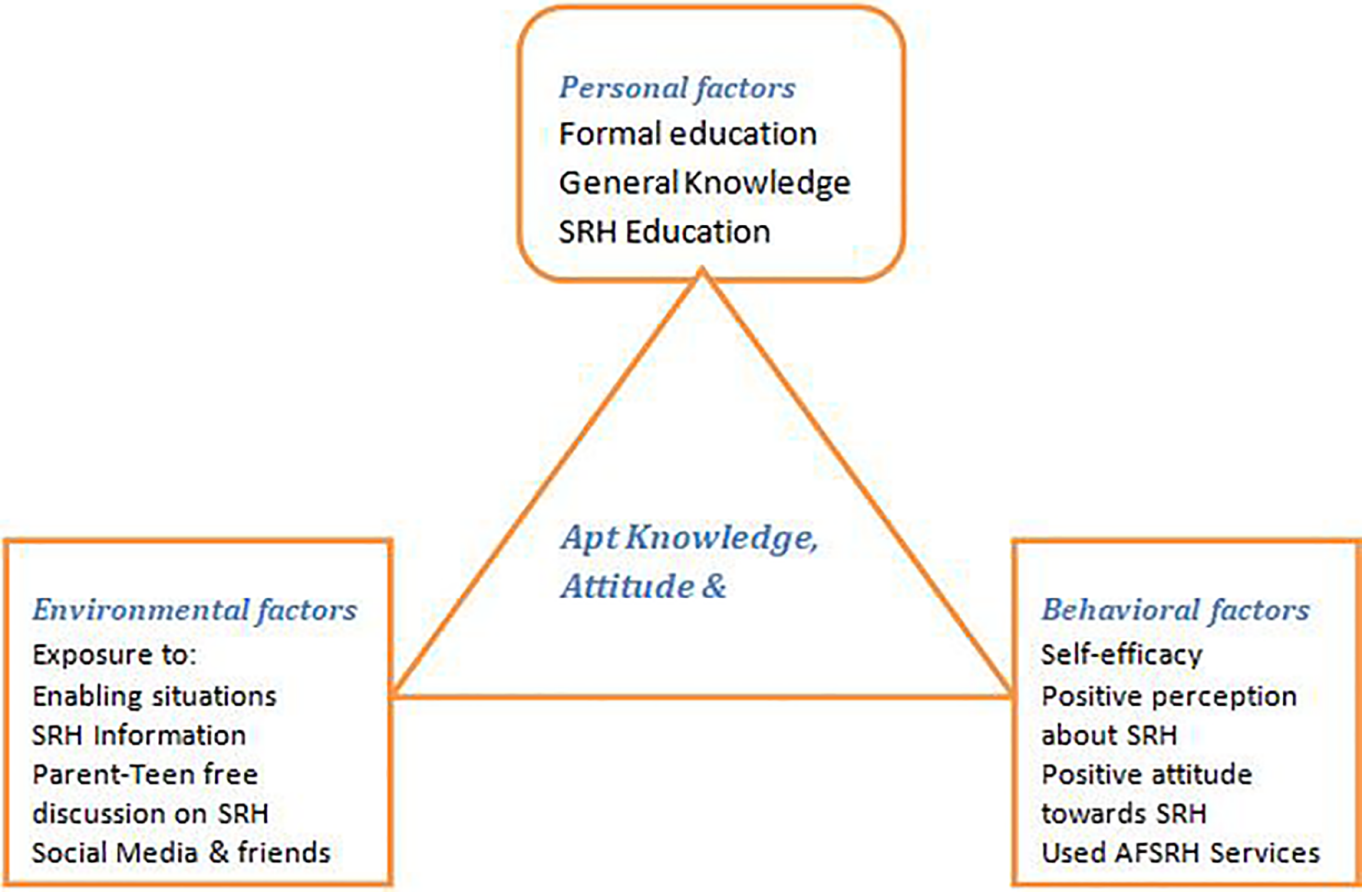
Sexual and reproductive health knowledge, attitudes, and practices conceptual framework based on bandura's social cognitive theory. High school adolescents, Guji zone, Ethiopia. Feb. 2022.

Adolescents are said to be utilizing the Adolescent Friendly Health Services when trained on SRH information, Counseled about SRH Matters, and utilized Adolescent Friendly Reproductive Health Services for at least six months. Practices were measured using eight items (e.g., Do you have need base and complete, friendly Health Services in one unit?) yes/one if the availability of compassionate and adolescent-friendly Health Services and no/zero if not. In Summary this study aimed to determine adolescents Knowledge, attitude and practice on Sexual and reproductive health among pastoral community. We compared the SRH knowledge, attitude and practice between the Gorodola high schools and Wadara high schools as case and compares. We identified Adolescents SRH KAP as the outcome variable and categorized the independent variables as personal, behavioral, and environmental-level factors. We used 34 items to measure appropriate SRH knowledge, attitude and practice in this study. Agree/one had appropriate information, knowledge, enabling situations, perceptions, and self-efficacy, practices and disagree/zero if not. The level of KAP was categorized using mean scores; thus, a knowledge score above the mean is termed appropriate KAP of SRH (see Figure 1).

### Data collection tool measurement and procedure

We employed a structured, self-administered questionnaire to collect data. The tool was developed in English and then translated into local languages. The questionnaire contained socio-demographic information on students and their parents, reproductive health-related characteristics of study participants, and SRH knowledge, attitude, and practice-related items. Five SRH experts reviewed the questionnaire's content and validity. The experts separately reviewed each item retained in the scale. Responding to an expert's recommendation, we modified the questionnaire and assessed its internal consistency. Fourteen diploma community nurses with prior data collection experience collected the data under the supervision of two public health officers.

### Data quality assurance

We distributed a pretested questionnaire to 5%) of the total sample. A translator translated all study instruments into local languages (Afan Oromo) and back-translated them into English to ensure the questions’ clarity, wording, and logical sequence. All data collectors and supervisors received a one-day intensive training on the study's purpose, methodologies, and data collection techniques. Based on the pretest results, we implemented all necessary changes to improve questionnaire completion. As a result of the pretest, confusing and lengthy questions were removed and shortened. The principal investigator and field supervisors closely monitored and coordinated the data collection process. We double-checked all data for completeness and consistency before analysis. We collected the data in a quiet area with study participants, away from noise or disturbance.

## Data analysis

After checking for completeness and internal consistency, the data were coded and entered into the Epi Data 3.1 computer software package. We exported the data to SPSS version 23 for further analysis. Descriptive statistics with frequency and mean + standard deviations (SDs) for continuous variables were used to describe the SRH-KAP of the adolescent school population. We used Chi-square and *T*-tests for analysis. Additionally, we employed bivariate linear regression to investigate the relationship between KAP and independent variables and potential confounders like age, religion, relationship status, and income generation. Multiple linear regression analyses examined respondents’ SRH knowledge, attitude, and practice. Analysis of Variance (ANOVA) values for overall SRH knowledge (F = 17.418, *p* < 0.001), SRH attitude (F = 16.757, *p* < 0.001), and SRH practice (F = 18.546, *p* < 0.001) indicated that our multiple linear regression model performed well and would be a good predictor of the primary outcome variables. Variables with *p* < 0.05 were considered statistically significant.

### Ethical consideration

We obtained ethical clearance from Jimma University (Ref. No. IHRPCA/721/202, date 17/08/2020) and the Bureau of Regional Health, Oromia (Lakk/Ref No. BEFO/MBTF/2081, date 27/01/2013, ETC. or 07/10/2020). Earlier in the in-depth interview and FGD, all study participants gave verbal informed consent.

Consent has been obtained from the ZHD, all district health offices, and education administrative centers to access schools and health facilities. Study participants were requested to consent in writing for their involvement in the Research, verbal and/or written consent followed a clear brief on the purpose of the study.

The study objectives were explained at each level, and informed consent was obtained from the parents of each study subject, a minor aged 14–18. The informant was required to consent on behalf of his or her involvement in the study. The study participants’ right to refuse at the beginning or interval was also respected.

The questionnaire did not require participants’ identification, including their names and addresses. All information has been strictly protected throughout the study period and respected by a researcher with the utmost care. All study participants were informed that participation was voluntary and that all the data would be managed confidentially with the utmost care. For instance, as part of privacy protection, data collectors were trained on the privacy of each study participant. The questionnaire sheet was mailed and packed immediately after being filled out by each study participant.

The findings of this Research have been disseminated to all concerned bodies. RH policymakers, the MCHD/FMoH, and all RHB can utilize them. The manuscripts have been published in reputable journals and presented at different conferences at national and international summits.

## Results

### Socio-demographic characteristics

Seven hundred seventy-three adolescents participated in the study with a response rate of 98.1%. About 49.7% of participants were from the case district (±0.5 SD). The mean age of the participants was 17.3 years with (±1.3 SD). The mean educational level of participants was 10.4463 (±1.1 SD), and 56% of participants came from Rural areas. Most participants, nearly 97.3%, were Oromo with (±0.2) (See Table 1).

**Table 1:** Socio-demographic characteristics of Gorodola and Wadara districts high schools adolescent students, Guji zone, Ethiopia. Feb. 2022.

Variables	Category	Case Schools *n* = 384		Compare Schools *n* = 389		Total *N* = 788, *n* = 773	
		*n*	%	*n*	%	*N*	(%)
Districts		384	49.7	389	50.3	773	98
Residence	Urban	145	37.8	195	50.1	340	44
	Rural	239	62.2	194	49.9	433	56
Age	Middle age adolescent	119	30.9	109	28	228	29.5
	Late age adolescents	265	68.9	280	72	545	70.5
Sex	Male	256	66.7	203	52.2	459	59.4
	Female	128	33.3	186	47.8	314	40.6
Marital status	Single	347	90.4	360	92.5	701	91.5
	Married	34	8.9	25	6.4	59	7.6
	Separated	3	0.8	4	1	7	0.9
Education	Grades 9 & 10	196	51	190	48.8	386	49
	Grades 11 & 12	188	49	199	51	387	49
Religions	Protestant	227	59.1	138	35.5	365	46.3
	Muslim	94	24.5	170	43.7	264	33.5
	Orthodox	18	4.7	57	14.7	75	9.5
	Others	45	11.4	24	6	69	8.8

### Knowledge

The finding showed that the proportion of SRH knowledge is 168(43.8%) among Gorodola high schools and Wadara high schools” in steady case and compares, 235(60.4%), at 47% of the mean SRH knowledge. The mean scores of the cases and comparisons were significant (mean diff. = −0.05182; *P* < 0.001; 95% CI = (−0.07457 to −0.029). The mean SRH Knowledge scores between the case and compares were significant (mean scores = 0.493 compared to 0.442 cases). We conducted multiple linear regressions to predict SRH knowledge. The study showed that for every additional learning risk of multiple partners, adolescent SRH knowledge increased by 0.071 (see Table 2).

**Table 2:** Proportion of SRH, knowledge, attitudes, and practices of significant covariates among adolescents of Gorodola and Wadara high schools, Guji zone, Ethiopia. Feb 2022.

Significant variables	Case (*n* = 384)	Compare (*n* = 389)	The proportion of diff.	Unstandardized coefficient. B	*P*-value
Percentage of SRH knowledge (±SD)					
Communicated SRH information.	38% (±0.486)	27.8% (±0.448)	10.2%	0.041	*P* < 0.001
Know Health Facilities that provide FHS	33.6% (±0.47)	36.5% (±0.482)	−2.9%	0.067	*P* < 0.001
Knowledge of the right to utilize FHS	57.8% (±0.49)	62.5% (±0.485)	−4.7%	0.058	*P* < 0.001
Knowledge of decision-making,	6.9% (±0.47)	82.8% (±0.378)	−21.3%	0.058	*P* < 0.001
Recognizing the risk of multiple partners	58.1% (±0.49)	58.4% (±0.494)	−0.3%	0.071	*P* < 0.001
Percentage of Attitudes (±SD)					
Choose FHS from other methods	65.6% (±0.48)	80.7% (±0.395)	−15.1%	0.135	*P* < 0.001
Utilize FHS without influence	36.2% (±0.48)	51.2% (±0.5)	−15%	0.161	*P* < 0.001
Freely discuss SRH issues with parents	44.8% (±0.5)	41.6% (±0.494)	3.2%	0.154	*P* < 0.001
Compassionate care to need-based FHS	47.9% (±0.5)	59.4% (±0.492)	−11.5%	0.166	*P* < 0.001
Percentage of practices (±SD)					
Regularly visited FHS	22.4% (±0.42)	30.6% (±0.461)	−8.2%	0.118	*P* < 0.001
Accesses complete, FHS in one unit?	18.8% (±0.4)	24.9% (±0.421)	−6.1%	0.105	*P* < 0.001

### Attitudes

The finding showed that the proportion of Adolescents’ attitudes toward SRH issues among the cases schools was 177(46.1%), compared to schools 234(60.2%), at 37% of the mean attitudes. The cases and compared mean scores have significance (−0.08568; *P* < 0.001; 95% CI = (−0.12 to −0.051). The mean scores of adolescent attitudes towards SRH Issues scores between the cases and compared schools were significant (0.519 compared to 0.4333 cases). We conducted multiple linear regressions to predict adolescents’ attitudes toward SRH issues. The study showed that for every additional care by healthcare providers, “adolescents” attitudes toward SRH issues are highly predicted to increase by 0.166 (see Table 2).

### Practice

The outcomes showed that the proportion of SRH services practices was 136(35.4%), compared to the compared group 214(55%), at 41% of the mean SRH practices. The mean scores of the case and compared were significant (mean diff. = −0.08169; *P* < 0.001; 95% CI = (−0.1 to −0.0559). The mean SRH practice scores between the two study arms were significant (mean scores = 0.4483 compared to 0.3666 cases). We conducted multiple linear regressions to predict SRH practices. The study demonstrated that every additional regular visit to FHS predicted an increase of 0.118 in adolescent SRH services practices (see Table 2).

## Discussion

The outcome indicated that the survey identified participants in the case and compared have low SRH knowledge, attitudes, and practice levels. In this research, we found that sexual and reproductive health education, regular visits to friendly health services, and receiving respectful care were more predictive of knowledge, attitudes, and SRH services utilization. It implied that adolescents’ SRH knowledge, attitude, and service utilization had been influenced by exposure to SRH Education, regularly visiting Friendly health services, and respectful services by Healthcare providers. The primary purpose of this assessment was to assess the level of SRH on KAP among high school adolescent students in the pastoral community of Guji Zone.

Our study found that the proportion of appropriate SRH knowledge was 44.2%. These findings were comparable with Melaku et al., Ethiopia, 43.5% (17), who discussed participants’ knowledge of contraceptive methods, and Kemigisha et al. (2018), Uganda, 47% (18). However, the finding was lesser than similar studies conducted in Arbamich, Ethiopia, 90.7% (19) et al., 2014) and Uganda, 95% (18). However, more significant than Olijira et al. (2013), 24.5% (4) showed that only one-fourth of -school adolescents have comprehensive HIV/AIDS knowledge. The proportion of SRH Knowledge showed that the compared arm was higher than the case group. This finding might be justifiable by explaining that most of the compared group consisted of urban residents. Thus, they might access more SRH info from different sources, friends, and Social media. This suggestion corresponds with Menna et al. (20), which showed that teenagers received SRH information from peers and media. For instance, Gupta et al. (21) discussed that urban adolescents are more knowledgeable about SRH Issues than rural adolescents. In our study, we observed that an increase in SRH knowledge is predicted by 0.1 for every additional learning risk of multiple sexual partners. This outcome is comparable with a study conducted in India (22), which discussed that repeatedly being exposed to learning opportunities has improved knowledge.

We compared the proportion of positive attitudes towards SRH matters, which was 60% in our study, with the 50% reported by Baku et al. (23) and by (24), both of which discussed favorable attitudes toward SRH matters. Our study showed a higher proportion compared to a study conducted by Airelobhegbe Dorcas Ehiaghe1 and Amadou Barrow (24.2%) (25) and a lower proportion compared to a study by Ayalew et al. (26) in Ethiopia (53.4%) (Ayalew et al.). However, contrary to the current study outcomes, Rehnström Loi et al., 53.2%, Kenya (27) and Mbadu Muanda et al. (28), DR. Congo (Muanda et al.) reported discussed negative attitudes towards SRH because of providers’ judgmental attitudes. Service providers’ positive attitudes toward SRH and their compassionate care could explain this phenomenon. This recommendation was consistent with Tilahun et al. (2018) and Mengistie et al. (15), who indicated that the other way round, providers’ optimistic attitudes had created positive attitudes in adolescents toward SRH matters.

The study found that providing additional care by healthcare providers’ friendly health services predicted an increase of 0.2 in adolescents’ attitudes toward SRH issues. This finding was comparable with Kennedy et al., who discussed how friendly providers had contributed to adolescents’ positive attitudes toward SRH service. Similarly, Ayalew et al. discussed that favorable attitudes are predictors of developing adolescents’ positive attitudes toward SRH issues (29, 26). On the other hand, Mbadu Muanda et al. (28) identified that the judgmental attitude of service providers harms adolescents’ attitudes toward SRH Issues (24, 25).

The proportion of SRH Services utilization among adolescents was 45%. This finding is comparable with research conducted in Ethiopia, Tlaye et al. (30), 33.8%, Ethiopia (Tlaye et al.) and South Ethiopia. 39.5% (31, 32) discussed at least one reproductive health service utilization. This figure is higher than a similar study conducted in southwest Ethiopia, 19.8% (Tilahun and Mamo) and Binu et al. (33), 21.2% (Binu et al.), and less than other similar studies conducted in Ethiopia. 41% (34), and Melaku et al., 85.8% (17). The study setting and cultural differences among pastoral communities against the urban setting, Awabel district, and Mekelle in northern Ethiopia may explain this discrepancy. This suggestion corresponds with Menna et al. (2022) (31), which showed that urban adolescents more utilize SRH Services than rural ones.

According to the linear regression analysis findings, every additional regular visit to FHS predicts an increase of 0.118 in adolescent-friendly health services practice. This finding was in line with Tilahun et al. (35), who discussed that visiting a health facility for other health services had been associated with an increase in SRH Services utilization [Tilahun et al. (35)], [Gebrehana et al. (36)]. Access to SRH information from providers during the visits might explain this phenomenon. This suggestion aligns with the findings of Rada Reproductive Health 2014 (37), which indicated that adolescents received SRH information from doctors or medics as a source.

### Strength and limitation

The study has both strengths and weaknesses. It is the first study to compare pastoral school adolescents of KAP on SRH in cases and compare urban and rural settings. It may have important policy implications for future SRH service improvement in the pastoral areas. Furthermore, we collected sufficient samples to discern differences between the two groups. Although we tried to minimize the study's flaws, readers should exercise caution when interpreting the findings. It is essential to acknowledge that every study comes with limitations. One of the limitations of this study was that respondents may be prone to social desirability biases, which may have contributed to underreporting some KAP on SRH, given the sensitive nature of the issues and the reliance on self-reports. This study's scope was limited to public high schools, making it challenging to generalize the findings to private schools and non-student adolescents in the study areas.

## Conclusion and recommendation

Overall, we found that participants possessed fairly middle-level SRH knowledge, positive attitudes towards SRH, and low practices; thus, Providing continued Reproductive health Information on the risks of multiple sexual partners, making adolescents friendly health services Compassionate, and providing proper SRH Information on every regular visit to health Services were the most predictors of SRH knowledge and positive attitudes towards SRH and practices.

The outcomes suggest that health facilities strengthen continued RH Information to adolescents on each regular friendly health service, Discouraging multi sexual partners and encouraging health sexual behavior, and motivate healthcare providers to deliver compassionate care. Therefore, health facilities authorities, school administrators, and relevant stakeholders should consider providing appropriate SRH information at each friendly health services visit.

## Data Availability

The original contributions presented in the study are included in the article/Supplementary Material, further inquiries can be directed to the corresponding author.
